# Weighted Multi-marker Genetic Risk Scores for Incident Coronary Heart Disease among Individuals of African, Latino and East-Asian Ancestry

**DOI:** 10.1038/s41598-018-25128-x

**Published:** 2018-05-01

**Authors:** Carlos Iribarren, Meng Lu, Eric Jorgenson, Manuel Martínez, Carla Lluis-Ganella, Isaac Subirana, Eduardo Salas, Roberto Elosua

**Affiliations:** 1 Kaiser Permanente Northern California Division of Research, Oakland, CA USA; 2 GenDiag, Inc/Ferrer in Code, Inc., Barcelona, Spain; 3 CIBER of Epidemiology and Public Health, Barcelona, Spain; 4 Cardiovascular Epidemiology and Genetics, IMIM, Barcelona, Spain; 5 CIBER of Cardiovascular Diseases (CIBERCV), Barcelona, Spain

## Abstract

We examined the clinical utility of two multi-locus genetic risk scores (GRSs) previously validated in Europeans among persons of African (AFR; n = 2,089), Latino (LAT; n = 4,349) and East-Asian (EA; n = 4,804) ancestry. We used data from the GERA cohort (30–79 years old, 68 to 73% female). We utilized two GRSs with 12 and 51 SNPs, respectively, and the Framingham Risk Score (FRS) to estimate 10-year CHD risk. After a median 8.7 years of follow-up, 450 incident CHD events were documented (95 in AFR, 316 in LAT and 39 EA, respectively). In a model adjusting for principal components and risk factors, tertile 3 vs. tertile 1 of GRS_12 was associated with 1.86 (95% CI, 1.15–3.01), 1.52 (95% CI, 1.02–2.25) and 1.19 (95% CI, 0.77–1.83) increased hazard of CHD in AFR, LAT and EA, respectively. Inclusion of the GRSs in models containing the FRS did not increase the C-statistic but resulted in net overall reclassification of 10% of AFR, 7% LAT and EA and in reclassification of 13% of AFR and EA as well as 10% LAT in the intermediate FRS risk subset. Our results support the usefulness of incorporating genetic information into risk assessment for primary prevention among minority subjects in the U.S.

## Introduction

The vast majority of cardiovascular genomic research has been conducted among populations of European origin. Therefore, subjects of African, Latino or Asian descent remain under-represented in this field^1^. Recently, we published a study showing improvement of the predictive capacity of the Framingham Risk Score after inclusion of four multi-locus genetic risk scores with increasing number of single nucleotide polymorphism (SNPs, namely GRS_8, GRS_12, GRS_36 and GRS_51) for incident coronary heart disease (CHD) among subjects of European ancestry in the Genetic Epidemiology Resource in Adult Health and Aging (GERA) cohort^2^. In this study, we expand the prior investigation by reporting the CHD predictive performance of GRS_12 and GRS_51 among persons of African (AFR), Latino (LAT) and East-Asian (EA) ancestry in the GERA cohort. Since results for GRS_8 were similar to GRS_12 and results for GRS-36 were similar to GRS_51, we focused on GRS_12 and GRS_51.

## Methods

### Study Cohort

This study utilized genome-wide genetic data available on the Genetic Epidemiology Resource in Adult Health and Aging (GERA) cohort of 110,266 adult male and female Kaiser Permanente of Northern California (KPNC) members. The cohort has been described in detail elsewhere^3^. In brief, the GERA cohort was formed by including all racial and ethnic minority participants in the larger cohort of the Research Program on Genes, Environment and Health (RPGEH) with saliva samples (19% of the total); the remaining participants were drawn randomly from White non-Hispanic participants (81% of the total). All RPGEH participants responded to a self-administered questionnaire in 2007/08 that included information on medical history, ancestry, health behaviors (smoking, alcohol consumption, diet, physical activity and reproductive history) and current weight and height. The study’s experimental protocol was approved by the Kaiser Foundation Research Institute Institutional Review Board and all study subjects gave informed consent. Of the 110,266 subjects, 97,858 had complete genetic data for estimating the GRSs. Among those, the following sequential exclusions were applied: 80,322 European ancestry subjects, 726 subjects with “Other” ancestry, 755 for being younger than 30 or older than 79, 492 for having prior CVD, and 4,340 for missing data on one or more Framingham Risk Score components, resulting in a final analytical cohort of 11,242 persons of non-white or minority descent. The sample included 2,089 African-Americans, 4,349 Latinos and 4,804 East Asians. The outcome of interest was incident CHD events, including hospital primary discharge diagnoses of myocardial infarction, angina (stable or unstable) or coronary revascularization procedures (coronary by-pass or percutaneous intervention) or death due to CHD through the end of 2016. The ICD-9 and ICD-10 codes used in event ascertainment are given in Supplemental Table 1.

### Genotyping

Genotyping was conducted at the Institute for Human Genetics, University of California San Francisco, using custom designed Affymetrix Axiom arrays^4,5^. To maximize genome-wide coverage of common and less common variants, four specific arrays were designed for individuals of Non-Hispanic White (EUR array; 674,516 SNPs), East Asian (EAS array; 713,008 SNPs), African (AFR array; 893,630 SNPs) and Latino (LAT array; 817,808 SNPs) ancestry. The genome-wide arrays gave high quality genotypes, with very high genotype call rates (average 99.7%) and SNP reproducibility (99.9%)^4–6^. Genotype imputation was done on a per-array basis using IMPUTE2 v2.2.2 and using 1k genomes (phase I integrated release, March 2012, with Aug 2012 chromosome-X update, a cosmopolitan reference panel with singletons removed) as the reference panel^7^.

### Selection of genetic variants and multi-locus risk score generation

We originally selected 51 SNPs previously identified to be associated with CHD^8^. Of the 51 SNPs, 14 were directly genotyped in the entire GERA cohort (Supplemental Table 2). For the 37 imputed SNPs, the imputation r^2^ was over 95% in 30 SNPs, between 90–85% in 3 SNPs and between 70–85% in 4 SNPs (rs11556924, rs17514846, rs2895811 and rs4773144). These 4 SNPs with imputation r^2^ < 0.85 were not included in the estimation of the GRS’s. Best guessed imputed genotypes were considered in the generation of the GRSs.

The multi-locus GRS’s were computed as the sum of the number of risk alleles across all genetic variants after weighting each one by its estimated effect size in the CARDIoGRAMplusC4D Consortium^8^. GRS_12 included 8 genetic variants associated with CAD but not with risk factors included in the classical risk functions (total, LDL or HDL cholesterol, blood pressure, smoking and diabetes) in accordance to the data available at GWAS catalog reviewed on August, 2010 plus 4 variants to incorporate the *ALOX5AP* haplotype. GRS_51 included 47 variants reported to be associated with CAD dependently and independently of their association with risk factors included in the classical risk functions, plus the 4 ALOX5AP SNPs. The *ALOX5AP* presents an haplotype, called haplotype B, that has been reported to be associated with coronary heart disease in different populations^9–12^. This haplotype consisted of rs10507391-A, rs93155050-A, rs17222842-G and rs17216473-A (these 4 SNPs were imputed). As the haplotype diversity could capture genetic variability associated risk better than individual genetic variants, and there were consistent data supporting the association between the *ALOX5AP* Haplotype B and coronary heart disease we included this haplotype variant in both GRSs (GRS_12 and GRS_51). No significant deviations from Hardy-Weinberg equilibrium were noted for any of the SNPs. We have verified in 1000 genomes SNAP (SNP Annotation and Proxy Search; https://www.broadinstitute.org/mpg/snap/ldsearch.php) that all 51 SNPs are independent (LD r ^2^ < 0.50). Weighted GRSs were calculated using the formula $GRS=\sum _{i=1}^{n}{\beta }_{i}\cdot SN{P}_{i}$ where *β*_*i*_ is the estimated effect size reported for each variant, *SNP*_*i*_ is the number of copies of each individual SNP evaluated (with values 0, 1 or 2) and *n* is number of SNPs. A weight of 0.131 was applied to the presence of the haplotype^11,13^. Ten-year CHD risk was estimated using the Framingham risk function described by Wilson *et al*.^14^. We did not use the more recently developed Pooled Cohorts Equations^15^ because they apply to all cardiovascular disease including stroke, whereas the CARDIoGRAMplusC4D Consortium focused on coronary artery disease^8^. Age, gender, education level, race/ethnicity, smoking status, alcohol consumption, body mass index and family history of heart disease were available from the self-completed RPGEH survey, systolic and diastolic blood pressures were obtained from primary care outpatient visits closest to the survey date and lipid panels and serum creatinine (closest to survey date) were obtained from the health plan laboratory database. Diabetes status was derived by cross-linkage with the KPNC diabetes registry. Hypertension and hypercholesterolemia treatment was ascertained using the Pharmacy Information Management System (PIMS) relying on prescription dispensing (at the time of the RPGEH survey or up to 2 years prior) of drugs falling into the corresponding therapeutic class. Estimation of glomerular filtration rate (GFR) was done using the Modification of Diet in Renal Disease Study (MDRD) formula^16^.

### Statistical Analyses

First, we obtained univariate descriptive statistics according to race/ethnicity. Next, we examined the distributional properties of each of the GRSs in subjects who went on to develop CHD with those who remained free of CHD and compare means using t-tests. Age-adjusted rates of incident CHD were calculated for race/ethnicity-specific tertiles of GRS_12 and GRS_51 using Poisson regression. We then tested the association of each GRS (both as a continuous variable in SD units [Model 1] and as tertiles [Model 2] with the lowest tertile as the referent group) with incident CHD using Cox proportional hazards models, with sequential adjustment for classical CHD risk factors and genetic ancestry. To correct for differences in genetic ancestry, we included ancestry principal components (PCs) in our analyses. To calculate the PCs, we used Eigenstrat v4.2 on each of the four race/ethnicity groups as previously described^3,17^. For the non-Hispanic Whites, the first 10 ancestry PCs were included in each regression model, while for the 3 other race/ethnicity groups, the first 6 ancestry PCs were included. Subjects were right-censored at different times depending on incident events, vital status or health plan membership status. Model “a” included only the GRSs and the PCs; Model “b” included the PCs plus the individual Framingham risk score variables (age, gender, total cholesterol, HDL-C, systolic and diastolic blood pressure, smoking status and diabetes); Model “c” included Model “b” covariates plus family history of heart disease and Model “d” included additional covariates that are not part of the Framingham risk score, namely education level, body mass index, anti-hypertensives, lipid lowering drugs and alcohol consumption. To test the proportionality of hazards assumption, we plotted Schoenfeld residuals against time and tested the interaction of each GRS with follow-up time. There was no visual evidence of departure from zero slope and none of the interactions were statistically significant (all p-values > 0.10). Therefore, there was no evidence that the proportionality of hazards assumption was violated. We also performed a fixed effects (because the groups were recruited from the same source population) meta-analysis of one SD increment of each GRS and of the effects of tertile 2 and tertile 3 across all three ethnic groups and tested for heterogeneity^18^. In addition, we used three different statistical approaches to assess the potential value of including the GRSs in risk prediction: a) the calibration (goodness-of-fit) of the models using the Hosmer–Lemeshow test (with 10 bins to define risk strata); b) the discriminative capacity of the model using the concordance index (Harrell’s C-statistic); and c) reclassification improvement using the net reclassification improvement (NRI) index and the integrated discrimination improvement (IDI) index^19^. For the assessment of reclassification improvement, we adopted the same four risk categories used in our prior study among subjects of European ancestry (low, intermediate-low, intermediate-high and high, with cut-off points 0–9.9%, 10–14.9%, 15–19.9%, ≥ 20%, respectively)^20^. We calculated the expected number of events at 10-years in each risk category using Kaplan–Meier estimates. A bootstrapping method was used to construct confidence intervals for IDI and NRI in order to account for uncertainty in the Kaplan–Meier estimates, as suggested by Steyerberg *et al*.^21^. To correct for bias in the NRI estimation among individuals with intermediate risk, we used the method proposed by Paynter and Cook^22^. To theoretically assess the effect of the GRSs in improving clinical utility and outcomes, we applied the concepts of number needed to treat to prevent 1 CHD event under two different scenarios: i) treat with statins all the individuals at intermediate risk (one stage screening); and, ii) treat only the up-reclassified subjects (two-stage screening)^23^. We assumed that statins would reduce the risk of major coronary events by 24 percent^24^. All statistical analyses were done in SAS version 9.4, Stata Release 15 and R software (version 3.4.1).

## Results

The main sociodemographic and clinical characteristics across the ethnic groups are shown in Table 1. The African-American sample was slightly older than the Latino and the East Asian samples. There was a greater proportion of females among Latinos than among the other two ethnic groups. Education level was highest among East Asians and lowest among Latinos. Current smoking was more common in African-Americans, and least common in East Asians. Alcohol abstinence was more frequent in East Asians, and high alcohol intake more frequent among African-Americans. The prevalence of diabetes, obesity, anti-hypertensive medication use and of low GFR were higher in African-Americans than in the other two ethnic groups. The estimated average 10-year Framingham Risk Score was 7 percent in African Americans and 6 percent in Latinos and East Asians. Self-report of family history of heart attack ranged from 21 percent in East Asians to 25 percent in Latinos.

**Table 1:** Characteristics of the GERA Cohort, Minority Subjects free of CVD at baseline.

Baseline Characteristics	African-American n = 2,089	Latinos n = 4,349	East Asians n = 4,804
Age at Survey, years (mean ± SD) and n (%)	57.1 ± 9.7	55.3 ± 10.2	55.6 ± 10
30–54	822 (39.3%)	2,066 (47.5%)	2,187 (45.5%)
55–64	724 (34.7%)	1,308 (30.1%)	1,570 (32.7%)
65–79	543 (26.0%)	975 (22.4%)	1,047 (21.8%)
Gender, n (%)			
Male	633 (30.3%)	1,192 (27.4%)	1,507 (31.4%)
Female	1,456 (69.7%)	3,157 (72.6%)	3,297 (68.6%)
Education level, n (%)			
Less than college	275 (13.2%)	1,138 (26.2%)	559 (11.6%)
College or higher	1,637 (78.4%)	2,831 (65.1%)	3,989 (83.0%)
Missing	177 (8.5%)	380 (8.7%)	256 (5.3%)
Smoking status, n (%)			
Never	1,239 (59.3%)	2,827 (65.0%)	3,721 (77.5%)
Former	683 (32.7%)	1,283 (29.5%)	937 (19.5%)
Current	167 (8.0%)	239 (5.5%)	146 (3.0%)
Alcohol consumption (drinks/week), n (%)	2.5 ± 5.1	2.9 ± 5.2	1.7 ± 3.8
Abstinence	1,069 (51.2%)	2,049 (47.1%)	3,032 (63.1%)
Low (<8 in men, <4 in women)	515 (24.7%)	1,119 (25.7%)	957 (19.9%)
Medium (8–21 in men, 4–14 in women)	310 (14.8%)	828 (19.0%)	500 (10.4%)
High (>21 in men; >14 in women)	45 (2.2%)	89 (2.0%)	32 (0.7%)
Missing	150 (7.2%)	264 (6.1%)	283 (5.9%)
Diabetes mellitus, n (%)	508 (24.3%)	846 (19.5%)	839 (17.5%)
Body mass index, kg/m^2^ (mean ± SD) and n (%)	29.7 ± 6.4	28.4 ± 6	24.7 ± 4.3
<18	10 (0.5%)	20 (0.5%)	68 (1.4%)
18–24.9	443 (21.2%)	1,232 (28.3%)	2,727 (56.8%)
25–29.9	702 (33.6%)	1,509 (34.7%)	1,382 (28.8%)
> = 30	815 (39.0%)	1,345 (30.9%)	454 (9.5%)
Missing	119 (5.7%)	243 (5.6%)	173 (3.6%)
Systolic blood pressure, mmHg (mean ± SD)	128.9 ± 15.6	124.9 ± 15.3	123.0 ± 15.7
Diastolic blood pressure, mmHg (mean ± SD)	76.8 ± 10	74.1 ± 9.9	73.6 ± 10.2
Anti-hypertensives, n (%)	1,187 (56.8%)	1,676 (38.5%)	1,853 (38.6%)
HDL-C, mg/dL (mean ± SD)	55.5 ± 15.1	53.6 ± 14.3	57.5 ± 15
Total Cholesterol, mg/dL (mean ± SD)	190.4 ± 38	195.3 ± 37.2	196.3 ± 36.1
Cholesterol lowering drugs, n (%)	778 (37.2%)	1,408 (32.4%)	1,553 (32.3%)
GFR, mL/min/1.73 m^2^, (mean ± SD) and n (%)	82.7 ± 19.3	80.2 ± 17.2	80.4 ± 16.9
<60	197 (9.4%)	399 (9.2%)	389 (8.1%)
60–90	1,175 (56.2%)	2,754 (63.3%)	3,082 (64.2%)
> = 90	676 (32.4%)	1,070 (24.6%)	1,161 (24.2%)
Missing	41 (2.0%)	126 (2.9%)	172 (3.6%)
Framingham Risk Score (median [IQR] and n (%)	6.9 (6.7)	6.0 (6.5)	5.6 (5.8)
Low (<10%)	1,497 (71.7%)	3,262 (75.0%)	3,791 (78.9%)
Intermediate-Low (10–15%)	332 (15.9%)	627 (14.4%)	597 (12.4%)
Intermediate-High (16–20%)	141 (6.7%)	249 (5.7%)	233 (4.9%)
High (>20%)	119 (5.7%)	211 (4.9%)	183 (3.8%)
Family history of angina/heart attack, n (%)			
No	1,552 (74.3%)	3,227 (74.2%)	3,750 (78.1%)
Yes	507 (24.3%)	1,089 (25.0%)	1,011 (21.0%)
Missing	30 (1.4%)	33 (0.8%)	43 (0.9%)

CVD denotes cardiovascular disease; GFR denotes glomerular filtration rate; IQR denotes interquartile range.

**Figure 1:**
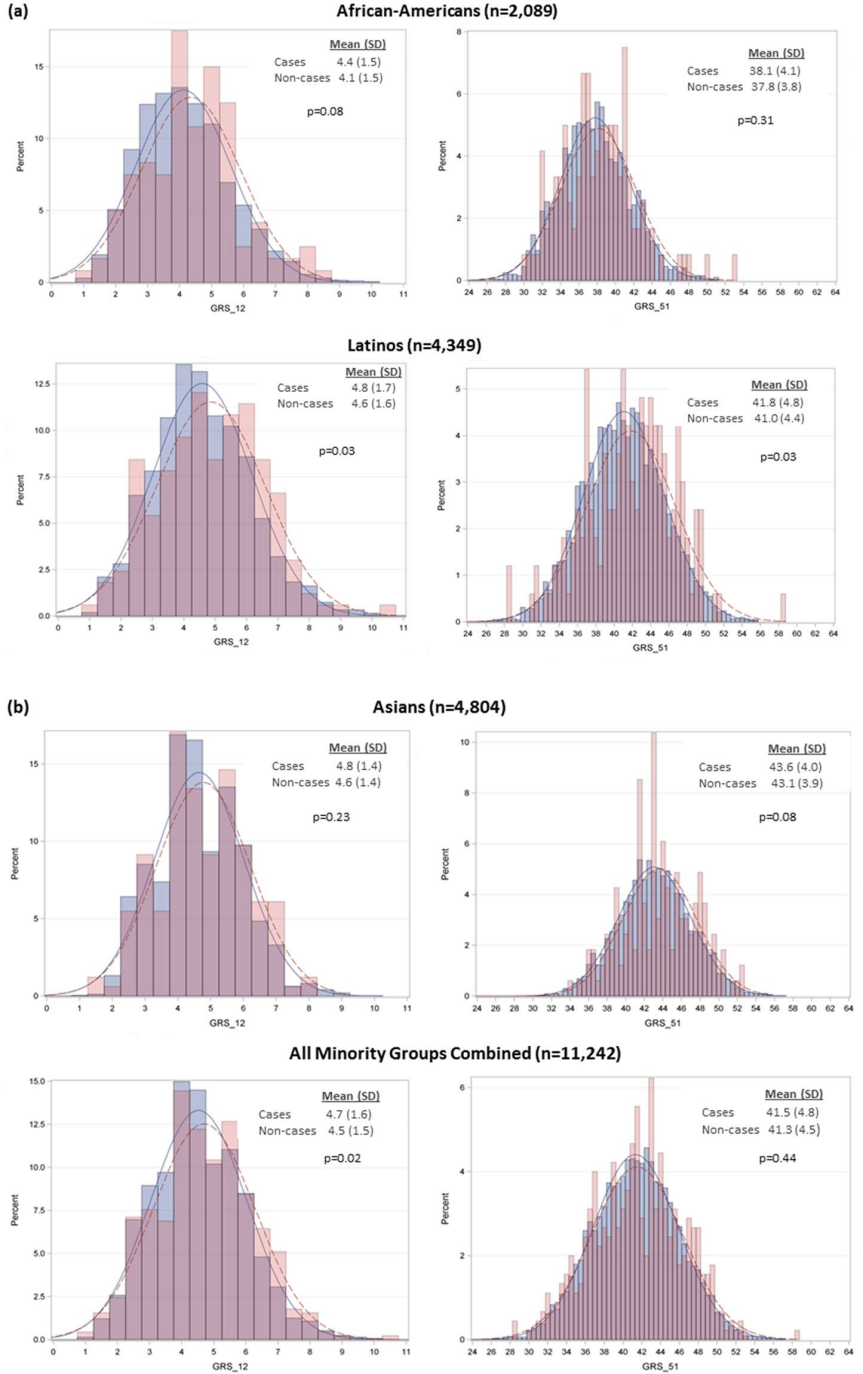
Distributional properties of GRS_12 and GRS_51 in the three race/ethnic groups. CHD cases are depicted in red, non-cases in blue.

The Pearson correlation between GRS_12 and GRS_51 was 0.62 in African-Americans and it was 0.59 in Latinos and East Asians. All GRSs means were higher in subjects with CHD events compared with subjects without CHD events, but the GRS_12 and GRS_51 means differ between CHD events and non-events in a statistically significant manner only in Latinos (both p = 0.03) (Fig. 1). The GRS_12 mean was also higher among CHD events than non-events when all minority groups were combined (p = 0.02).

**Figure 2:**
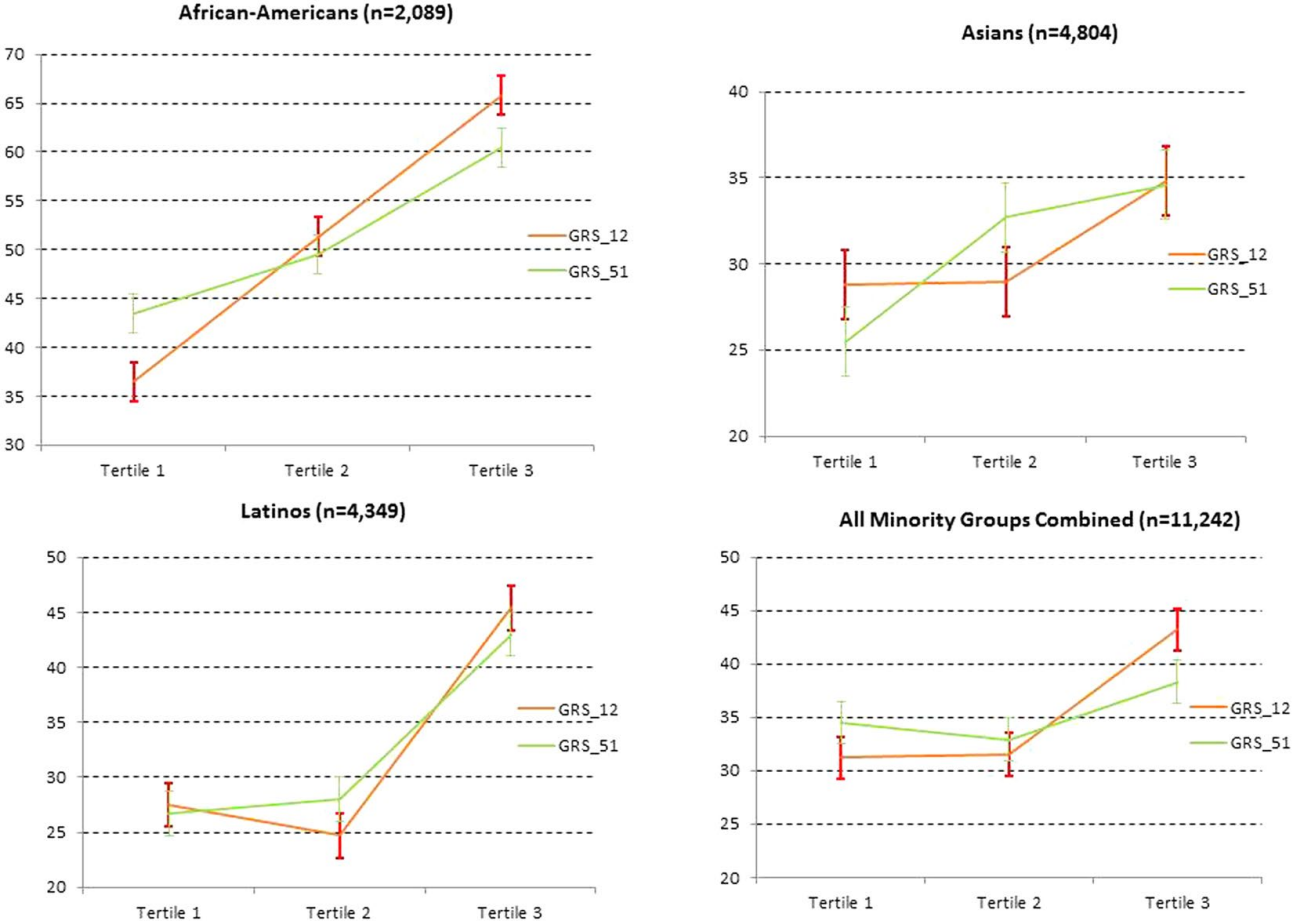
Age-adjusted rates of incident CHD according to quintiles of GRS_12 and GRS_51. Bars denote standard errors.

After a mean (SD) follow-up time of 8.7 (2.0) years, 450 incident CHD events were documented (95 in African-Americans, 316 in Latinos and 39 in East Asians). For both GRSs, there was a monotonic association across tertiles of GRSs and age-adjusted rates of incident CHD among African-Americans and East Asians. In Latinos, we observed a monotonic association for GRS_51 and a V-shape association for GRS_12 (Fig. 2).

**Figure 3:**
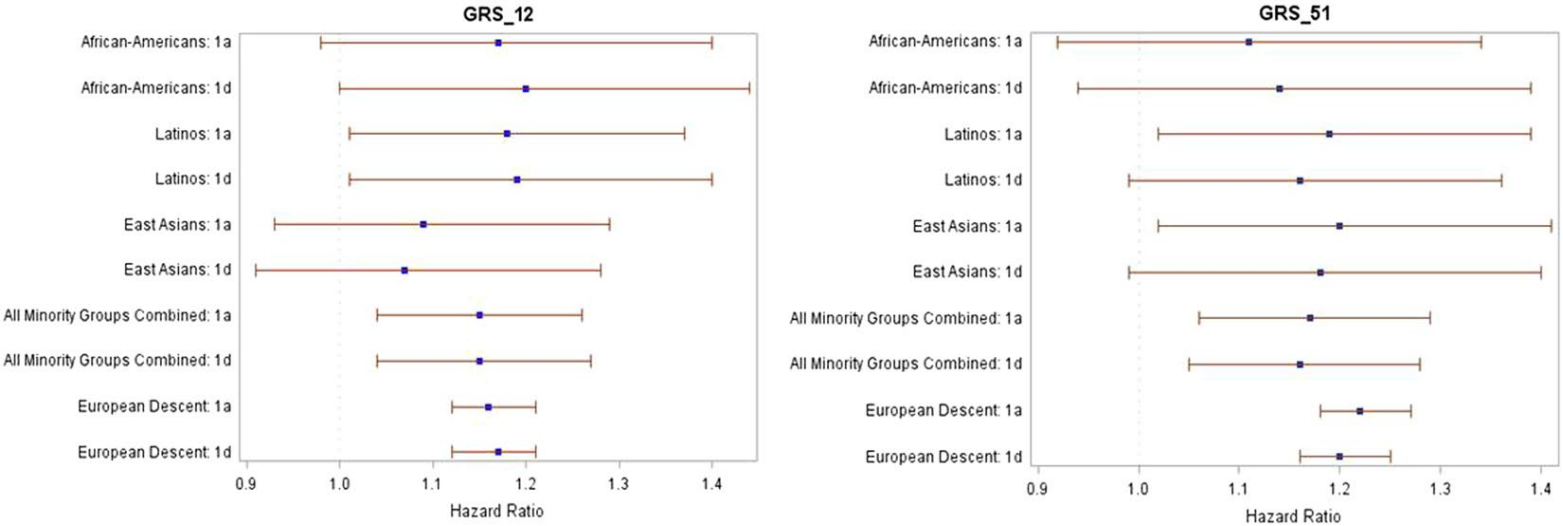
Forest plot for 1 SD increment of GRS in Model 1a (adjusted for PCs only) and 1d (fully-adjusted).

The results of the Cox regression analyses in each minority group and in the meta-analyses of all minority groups combined are shown in Table 2. GRS_12 and GRS_51 were associated with incident CHD in Latinos, only GRS_12 was associated with CHD in African-Americans and only GRS_51 was associated with CHD in East Asians when considering GRS tertiles. Forest plots comparing the effect of 1 SD increment in both GRS_12 and GRS_51 among the minority ethnic groups and the European subset are shown in Fig. 3 (to include the data from the European sample, we repeated the analysis previously published extending the follow-up time through the end of 2016). In the meta-analysis there was no heterogeneity across the minority groups and both GRSs were associated with CHD risk with a similar effect size. The strength of independent association of all the individual risk factors with incident CHD is shown in Supplemental Table 3.

**Table 2:** Association between genetic risk scores (GRSs) and incident CHD among GERA minority subjects.

	GRS_12	GRS_51
	African-Americans (n = 2,089)	
Model 1a	1.17 (0.98–1.40)	1.11 (0.92–1.34)
Model 1b	1.16 (0.97–1.39)	1.09 (0.90–1.32)
Model 1c	1.17 (0.97–1.40)	1.09 (0.90–1.32)
Model 1d	1.20 (1.00–1.44)	1.14 (0.94–1.39)
Model 2a		
Tertile 2	1.33 (0.82–2.17)	1.14 (0.71–1.82)
Tertile 3	1.81 (1.14–2.88)	1.42 (0.90–2.23)
Model 2b		
Tertile 2	1.33 (0.81–2.18)	1.06 (0.66–1.70)
Tertile 3	1.78 (1.12–2.83)	1.34 (0.85–2.11)
Model 2c		
Tertile 2	1.33 (0.82–2.18)	1.06 (0.66–1.70)
Tertile 3	1.78 (1.12–2.84)	1.34 (0.85–2.11)
Model 2d		
Tertile 2	1.37 (0.82–2.29)	1.04 (0.63–1.71)
Tertile 3	1.86 (1.15–3.01)	1.49 (0.93–2.39)
	**Latinos (n = 4,349)**	
Model 1a	1.18 (1.01–1.37)	1.19 (1.02–1.39)
Model 1b	1.23 (1.05–1.43)	1.19 (1.02–1.39)
Model 1c	1.23 (1.05–1.44)	1.19 (1.02–1.38)
Model 1d	1.19 (1.01–1.40)	1.16 (0.99–1.36)
Model 2a		
Tertile 2	0.94 (0.62–1.41)	0.99 (0.65–1.48)
Tertile 3	1.59 (1.09–2.31)	1.61 (1.12–2.34)
Model 2b		
Tertile 2	0.89 (0.59–1.34)	1.06 (0.70–1.60)
Tertile 3	1.68 (1.15–2.45)	1.54 (1.06–2.24)
Model 2c		
Tertile 2	0.88 (0.58–1.33)	1.07 (0.71–1.62)
Tertile 3	1.68 (1.15–2.45)	1.55 (1.07–2.25)
Model 2d		
Tertile 2	0.89 (0.58–1.37)	1.06 (0.69–1.62)
Tertile 3	1.52 (1.02–2.25)	1.40 (0.95–2.06)
	**Asians (n = 4,804)**	
Model 1a	1.09 (0.93–1.29)	1.20 (1.02–1.41)
Model 1b	1.10 (0.93–1.30)	1.24 (1.05–1.46)
Model 1c	1.10 (0.94–1.30)	1.23 (1.04–1.46)
Model 1d	1.07 (0.91–1.28)	1.18 (0.99–1.40)
Model 2a		
Tertile 2	0.87 (0.57–1.33)	1.33 (0.87–2.03)
Tertile 3	1.19 (0.79–1.79)	1.48 (0.98–2.25)
Model 2b		
Tertile 2	0.95 (0.62–1.46)	1.36 (0.89–2.09)
Tertile 3	1.27 (0.84–1.92)	1.57 (1.04–2.39)
Model 2c		
Tertile 2	0.96 (0.63–1.47)	1.34 (0.88–2.06)
Tertile 3	1.27 (0.84–1.92)	1.56 (1.02–2.36)
Model 2d		
Tertile 2	1.01 (0.65–1.57)	1.37 (0.88–2.12)
Tertile 3	1.19 (0.77–1.83)	1.43 (0.93–2.22)
	**Fixed-effects Meta-analysis of All Minority Groups Combined (n = 11,242)**	
Model 1a	1.15 (1.04–1.26)	1.17 (1.06–1.29)
Model 1b	1.17 (1.06–1.28)	1.18 (1.07–1.30)
Model 1c	1.17 (1.06–1.28)	1.18 (1.07–1.30)
Model 1d	1.15 (1.04–1.27)	1.16 (1.05–1.28)
Model 2a		
Tertile 2	1.00 (0.78–1.29)	1.14 (0.89–1.47)
Tertile 3	1.49 (1.18–1.89)	1.52 (1.20–1.92)
Model 2b		
Tertile 2	1.01 (0.78–1.30)	1.16 (0.90–1.49)
Tertile 3	1.55 (1.22–1.97)	1.49 (1.18–1.89)
Model 2c		
Tertile 2	1.01 (0.79–1.31)	1.15 (0.90–1.48)
Tertile 3	1.55 (1.22–1.97)	1.49 (1.18–1.89)
Model 2d		
Tertile 2	1.04 (0.80–1.36)	1.15 (0.89–1.50)
Tertile 3	1.48 (1.15–1.90)	1.43 (1.12–1.84)

CHD denotes coronary heart disease;

Table entries are hazard ratios (95% CI);

See Statistical Methods for explanation of Models 1a-1d and 2a-2d specifications.

The Harrell C-statistic, indicative of the area under the curve (AUC) after the addition of the GRSs to models already containing the FRS, changed only marginally, and the p-values for the difference in the AUC were not statistically significant (all > 0.10) (Table 3). The results of all the Hosmer-Lemeshow tests showed improved goodness fit of the models after including the GRSs in the equation (all p-values ≥ 0.07) (Table 3). Except in East Asians for both GRS_12 and GRS_51, there were clear improvements in the discrimination slope (IDI) of the updated models. The reclassification of individuals after including GRS_12 ranged between 7 and 10 percent overall and between 10 and 13 percent in the intermediate risk subset across minority groups, and was 6 percent overall and 9 percent in the intermediate risk subset among all minority groups combined. Notably, the contribution to reclassification was mainly from subjects who went on to develop CHD. Reclassification of individuals after including GRS_51 ranged between 2 and 6 percent overall and between 0 and 9 percent in the intermediate risk subset, and was 3 percent overall and 4 percent in the intermediate risk subset among all minority groups combined. The reclassification tables are provided in Supplemental Table 4a–d. Based on the theoretical efficacy of statins (24% reduction in CHD incidence)^20^, we estimated the number of CHD events that would be prevented by the systematic treatment with statins to all the subjects in the intermediate group (“one stage screening”) in relation to those prevented by treating the up-reclassified subject using GRS_12 or GRS_51 (Table 4 and Supplemental Table 4a–d). For GRS_12, the two-stage approach was 1.4, 3.3, 2.0 and 2.9 more efficient that the one-stage approach in terms of individuals needed to treat to prevent one event, in African-Americans, Latinos, Asians and all minority groups combined, respectively. For GRS_51 the two-stage approach was 2.8, 3.8, 1.6 and 3.8 more efficient that the one-stage approach in terms of individuals needed to treat to prevent one event, in African-Americans, Latinos, Asians and all minority groups combined, respectively.

**Table 3:** Model Calibration, Discriminative Capacity and Reclassification for Incident CHD among GERA minority subjects.

	GRS_12	GRS_51
	African-Americans (n = 2,089)	
Harrell C Statistic		
Model with FRS	0.687	0.687
Model with FRS + GRS	0.691	0.690
P value for difference	0.57	0.45
Hosmer-Lemeshow Chi-Square		
Model with FRS	9.7 (9); 0.38	7.8 (9); 0.55
Model with FRS + GRS	11.3 (9); 0.26	6.2 (9); 0.72
P value for difference		
Integrated Discrimination Improvement (IDI)		
All Cohort	0.18 (0.00–0.97)	0.06 (−0.03–0.63)
Intermediate Risk Subset	0.31 (−0.07–1.53)	0.18 (−0.09–1.68)
Category-based Net Reclassification Index (NRI) in the Full Cohort		
Subjects with CHD events	0.11 (0.00–0.22)	0.02 (−0.05–0.10)
Subjects without CHD events	−0.01 (−0.02–0.01)	−0.01 (−0.02–0.00)
All subjects	0.10 (−0.01–0.21)	0.02 (−0.06–0.09)
Bias-corrected Category-based Net Reclassification Index (NRI) in the Intermediate Risk Subset		
Subjects with CHD events	0.15 (0.02–0.28)	0.03 (−0.07–0.12)
Subjects without CHD events	−0.02 (−0.07–0.03)	−0.02 (−0.06–0.01)
All subjects	0.13 (−0.01–0.27)	0.00 (−0.09–0.10)
	**Latinos (n = 4,349)**	
Harrell C Statistic		
Model with FRS	0.714	0.714
Model with FRS + GRS	0.717	0.715
P value for difference	0.54	0.86
Hosmer-Lemeshow Chi-Square		
Model with FRS	6.1 (9); 0.73	6.1 (9); 0.73
Model with FRS + GRS	8.6 (9); 0.48	7.2 (9); 0.62
Integrated Discrimination Improvement (IDI)		
All Cohort	0.16 (0.01–0.69)	0.14 (−0.01–0.65)
Intermediate Risk Subset	0.39 (−0.03–2.22)	1.31 (0.14–5.01)
Category-based Net Reclassification Index (NRI) in the Full Cohort		
Subjects with CHD events	0.07 (−0.01–0.16)	0.06 (−0.03–0.15)
Subjects without CHD events	0.00 (−0.01–0.01)	−0.01 (−0.02–0.00)
All subjects	0.07 (−0.01–0.16)	0.05 (−0.04–0.14)
Bias-corrected Category-based Net Reclassification Index (NRI) in the Intermediate Risk Subset		
Subjects with CHD events	0.12 (−0.04–0.29)	0.09 (−0.06–0.23)
Subjects without CHD events	−0.03 (−0.06–0.01)	−0.04 (−0.07 – −0.00)
All subjects	0.10 (−0.06–0.26)	0.05 (−0.10–0.20)
	**Asians (n = 4,804)**	
Harrell’s C Statistic		
Model with FRS	0.745	0.745
Model with FRS + GRS	0.747	0.750
P value for difference	0.25	0.11
Hosmer-Lemeshow Chi-Square		
Model with FRS	4.4 (9); 0.88	4.4 (9); 0.89
Model with FRS + GRS	6.2 (9); 0.72	8.6 (9); 0.47
Integrated Discrimination Improvement (IDI)		
All Cohort	−0.01 (−0.06–0.18)	−0.01 (−0.11–0.19)
Intermediate Risk Subset	0.04 (−0.14–0.88)	−0.01 (−0.07–0.69)
Category-based Net Reclassification Index (NRI) in the Full Cohort		
Subjects with CHD events	0.08 (0.02–0.13)	0.06 (−0.02–0.13)
Subjects without CHD events	0.00 (−0.01–0.01)	0.00 (−0.01–0.01)
All subjects	0.07 (0.02–0.13)	0.06 (−0.02–0.13)
Bias-corrected Category-based Net Reclassification Index (NRI) in the Intermediate Risk Subset		
Subjects with CHD events	0.12 (0.02–0.21)	0.09 (−0.02–0.19)
Subjects without CHD events	0.01 (−0.02–0.03)	0.00 (−0.03–0.03)
All subjects	0.13 (0.03–0.22)	0.09 (−0.02–0.20)
	**All Minority Groups Combined (n = 11,242)**	
Harrell C Statistic		
Model with FRS	0.723	0.723
Model with FRS + GRS	0.725	0.723
P value for difference	0.25	0.76
Hosmer-Lemeshow Chi-Square		
Model with FRS	16.0 (9); 0.07	16.0 (9); 0.07
Model with FRS + GRS	7.8 (9); 0.56	9.6 (9); 0.38
Integrated Discrimination Improvement (IDI)		
All Cohort	0.07 (0.00–0.25)	0.01 (−0.01–0.13)
Intermediate Risk Subset	0.16 (−0.00–0.58)	0.06 (−0.01–0.59)
Category-based Net Reclassification Index (NRI) in the Full Cohort		
Subjects with CHD events	0.07 (0.02–0.11)	0.03 (−0.00–0.06)
Subjects without CHD events	0.00 (−0.01–0.00)	0.00 (−0.00–0.00)
All subjects	0.06 (0.02–0.11)	0.03 (−0.00–0.06)
Bias-corrected Category-based Net Reclassification Index (NRI) in the Intermediate Risk Subset		
Subjects with CHD events	0.10 (0.04–0.16)	0.04 (−0.01–0.09)
Subjects without CHD events	−0.01 (−0.03–0.01)	0.00 (−0.01–0.01)
All subjects	0.09 (0.03–0.16)	0.04 (−0.01–0.09)

CHD denotes coronary heart disease; FRS denotes Framingham Risk Score; GRS denotes genetic risk score.

**Table 4:** Clinical Utility Parameters for Incident CHD for the Two GRS among GERA Subjects of Minority Descent Classified as Intermediate Risk.

	Individuals to be treated (A)	Events predicted at 10 years (B)	Events potentially prevented (C = B*0.24)	Individuals needed to treat to prevent 1 CHD event (D = A/C)	Efficiency of the two-stage vs. one-stage approach
	**African-Americans (n = 2,089)**				
One-stage Screening	473	41	9.8	47	1
Two-stage Screening					
GRS_12	33	4	1.0	33	1.4 (47/33)
GRS_51	12	3	0.7	17	2.8 (47/17)
	**Latinos (n = 4,349)**				
One-stage Screening	876	48	11.5	73	1
Two-stage Screening					
GRS_12	31	6	1.4	22	3.3 (73/22)
GRS_51	39	9	2.2	19	3.8 (73/19)
	**Asians (n = 4,804)**				
One-stage Screening	830	46	11.0	75	1
Two-stage Screening					
GRS_12	19	2	0.5	38	2.0 (75/38)
GRS_51	24	2	0.5	48	1.6 (75/48)
	**All Minority Groups Combined (n = 11,242)**				
One-stage Screening	2179	135	32.4	68	1
Two-stage Screening					
GRS_12	59	11	2.6	23	2.9 (68/23)
GRS_51	30	7	1.7	18	3.8 (68/18)

CHD denotes coronary heart disease; GRS denotes genetic risk score.

## Discussion

This study extends our prior report examining the utility of multi-locus GRSs in CHD risk stratification among subjects of European ancestry in the GERA cohort^2^ to participants of African-American, Latino and East Asian ancestry. As noted in the European sample, a weighted GRS consisting of 12 autosomal genetic variants was significantly associated with incident CHD independently of risk factors and self-reported family history of heart disease among African-Americans and Latinos, but not among East Asians. In the meta-analysis adjusting for genetic diversity (PCs), risk factors and combining all three minority groups, subjects in the top tertile of GRS_12 had a 48 percent increased risk of CHD compared to subjects in the bottom tertile. In race/ethnic-specific models, the risk in the top tertile was more marked among African-Americans than among Latinos or East Asians. Furthermore, the inclusion of GRS_12 on top of the Framingham risk score provided up-reclassification between 6 percent (in analysis combining all minority groups) and 10 percent (in African-Americans) when considering the full cohort and between 9 percent (in analysis combining all minority groups) and 13 percent (in African-Americans and East Asians) when considering only intermediate risk subset. By comparison, in the European GERA sample, the NRI for GRS_12 was 5 percent in the full cohort and 9 percent in the intermediate risk subset^2^.

For the GRS enriched with 51 autosomal loci, we observed an independent statistically significant association only in the model comparing the upper with the lowest tertile among Latinos. In the meta-analysis adjusting for genetic diversity (PCs), risk factors and combining all three minority groups, subjects in the top tertile of GRS_51 had a 43 percent increased risk of CHD compared to subjects in the bottom tertile. The inclusion of GRS_51 on top of the Framingham risk score provided up-reclassification between 2 percent (in African-Americans) and 6 percent (in East Asians) when considering the full cohort and between 0 percent (in African-Americans) and 9 percent (in East Asians) when considering only intermediate risk subset. In the European GERA sample, the NRI for GRS_51 was 4 percent in the full cohort and 7 percent in the intermediate risk subset^2^.

There is substantial evidence supporting the usefulness of multi-locus GRSs for prediction of CHD in white populations^20,25–34^. Furthermore, prior studies indicate that the majority of the genetic effect operates independently of traditional risk factors^2,28,29^. On the other hand, there is a paucity of research focusing on GRSs for CHD among minority populations and published studies tend to be based on small population samples. Larifla *et al*. studied 537 Afro-Caribbean individuals (178 CHD cases and 359 controls) and found that a 19-SNPs GRS was a strong predictor of CHD^35^. Gui *et al*. performed a case-control study with 1,146 CHD cases and 1,146 controls recruited from 3 hospitals in the Hubei province, China^36^, and a 10-SNP GRS was associated with increased CHD risk after adjustment for traditional risk factors and improved risk prediction for CHD when assess by NRI and IDI. In a Pakistani case-control study (321 cases and 228 controls), the mean gene score for a 13-SNP GRS was significantly higher in cases than in controls^37^. The study by Qi *et al*. in 1,898 myocardial infarction (MI) cases and 2,096 population-based controls recruited from a Hispanic Costa Rican population showed that addition of a GRS calculated using the 3 SNPs showing the strongest association with MI improved discrimination of MI status^38^. Latinos in the US are genetically admixed, with a large amount of European ancestry and a small amount of Native and African ancestry^39^.

Current guidelines do not endorse incorporating genetic markers into cardiovascular risk prediction for primary prevention^40^. For example, use of DNA-based tests for cardiovascular risk assessment is not recommended by the most recent European Society of Cardiology guidelines (Class III, Level B recommendation)^41^. The three arguments behind this recommendation are: 1) As the variants have been identified in GWAS studies among European-ancestry subjects, those variants may not be relevant to individuals of other racial and ethnic backgrounds; 2) There is a lack of agreement regarding which genetic variants should be included and how genetic risk scores should be calculated; and 3) There are uncertainties about the improvement of the cardiovascular risk prediction and cardiovascular care by using genetic variants.

The current study provides new evidence addressing the first argument (i.e., whether the variants that have been identified in European ancestry enriched GWAS are also relevant to individuals of other racial and ethnic backgrounds). Our findings agree with previous studies reporting that most variants identified from GWAS in EA populations generalize to non-EA populations, although a significant proportion of these variants present different effect sizes in other ethnic groups^42–44^. These differences could be related to differential linkage disequilibrium patterns, differential genetic background of CHD and environmental factors exposure variability across ethnic groups. The lack of association between the GRS_51 and CHD risk in African-Americans could be explained by a dilution effect when including a higher number of variants that has been described in previous studies mainly affecting this ethnic group^45,46^. The lack of association between the GRS_12 and CHD in Asians should be considered with caution due to the low number of coronary events observed in this population.

With regard to the second argument (which genetic variants to include?), we used a strategy commonly used for combining multiple genetic variants^40^. The genetic variants for GRS_12 were selected from those identified in the initial GWAS studies using a selection process focused on the selection of genetic loci with direct association with overall cardiovascular risk^47^ with latter addition of a variant in LPA gene^20^ and the ALOX5AP B haplotype^2^, and not related to classical cardiovascular risk factors. On the other hand, GRS_51 includes variants associated with CHD independently of their association with standard cardiovascular risk factors. To create the multivariable GRS for each study participant we used the commonly accepted weighted method according to the beta value attributed to the each genetic variant by the CARDIoGRAMplusC4D Consortium^8^, assuming each genetic variant to be independently associated with risk according to an additive genetic model. For each genetic variant included in the GRS calculation, weightings of 0, 1 and 2 were attributed according to the number of risk alleles present. The GRSs we used have been successfully evaluated following the criteria for evaluation of novel markers of cardiovascular risk published as an AHA Scientific Statement^48^.

With regard to the third argument (uncertainties about the improvement of the cardiovascular risk prediction and cardiovascular care), our data demonstrate that the incorporation of the GRSs into the classical risk equations may reclassify up to 10 percent of the population categorized as intermediate risk. The population categorized as intermediate risk is “on the fence” that is, it is unclear whether an aggressive or a conservative approach is warranted. In those cases, patients up reclassified would be candidates for more aggressive management. Moreover, to assess clinical utility of the GRSs, we estimated the efficiency of the two-stage screening vs. one stage screening as the ratio of number of individuals needed to treat to prevent 1 CHD treating all subjects in the Framingham intermediate risk group to the number of individuals needed to treat to prevent 1 CHD among subjects up-reclassified to high risk group by incorporating genetic information. For GRS_12, the efficiency was 2.9 overall, and ranged from 1.4 in African-Americans to 3.3 in Latinos. For GRS_51, the efficiency was 3.8 overall, and ranged between 1.6 in East Asians to 3.8 in Latinos. In the European sample, the efficiencies of GRS_12 and GRS_51 were 1.9 and 1.6, respectively^49^.

Our study has some limitations. First, our sample sizes, particularly African-Americans (n = 2,089), were not large thus reducing statistical power. Moreover, the number of CHD events specially in the Asian population was limited affecting also our statistical power. Second, we did not have a full 10-year follow-up period, so 10-year CHD risk was extrapolated from the available average 8.7 years of follow-up. Third, our cohort subjects were all members of an integrated health care delivery system, thus our findings may not be generalizable to uninsured populations. Fourth, we used SNPs and weighting factors derived from European subjects. Future work ought to focus on deriving ethnic-specific GRSs with ethnic-SNPs validated across cohorts and ethnic-specific weighting factors that do not currently exist. A GWAS in 8,090 African Americans from 5 population-based cohorts replicated 17 loci previously associated with CHD in Caucasians, but found no novel variants among African-Americans^46^. Similarly, the PAGE Study that used the MetaboChip in 8,201 African-Americans in two U.S. cohorts was not able to replicate any loci in three additional cohorts^50^. Our study has also some strengths, including the fact that our minority subjects came from a single large “parent” cohort (the GERA cohort), so we did not have to pool or meta-analyze data from different cohorts and settings thus enhancing internal validity. Also, our cohort represents the experience of real-world contemporary clinical practice setting.

In conclusion, our analysis indicates that the predictive added value of GRSs generated in European ancestry populations persists in other ethnic groups, more clearly so in African-American and Latinos than in East Asians. This could represent true heterogeneity of causal effect across ancestries and implies that an optimization of GRSs for Asian populations is warranted.

What are the clinical implications of our findings? We agree with the guidelines that with the available information, universal use of DNA-based test for cardiovascular risk assessment should not be recommended. However, our results and the accumulated evidence support the use of the GRS in specific populations, namely those with intermediate risk for whom more aggressive therapy may have added benefit.

## Electronic supplementary material

Supplementary Material
