# Supplementary material for: Weighted Multi-marker Genetic Risk Scores for Incident Coronary Heart Disease among Individuals of African, Latino and East-Asian Ancestry
Source: Sci Rep. 2018 May 1;8:6853. doi: 10.1038/s41598-018-25128-x (PMC5931622; doi:10.1038/s41598-018-25128-x)
Supplement: Supplementary file 1 — Supplementary Material [file 41598_2018_25128_MOESM1_ESM.pdf]

Supplementary Material

Weighted Multi-marker Genetic Risk Scores for Incident Coronary Heart Disease among Individuals of African, Latino and East-Asian Ancestry

The Kaiser Permanente Genetic Epidemiology Resource in Adult Health and Aging (GERA) Cohort

Carlos Iribarren, MD, MPH, PhD[1]
Meng Lu, MD, MS[1]
Eric Jorgenson, PhD[1]
Manuel Martinez, BSc[2]
Carla Lluis-Ganella, MSc, PhD[2]
Isaac Subirana, MSc, PhD[3,4]
Eduardo Salas, MD, PhD[2]
Roberto Elosua, MD, PhD[4,5]

Authors Affiliations:

[1] Kaiser Permanente Northern California Division of Research, Oakland, CA (C.I., M.L., E.J.)
[2] GenDiag, Inc / Ferrer in Code, Inc., Barcelona, Spain (M.M., E.S., C.L-G.)
[3] CIBER of Epidemiology and Public Health, Barcelona, Spain (I.S.)
[4] Cardiovascular Epidemiology and Genetics, IMIM, Barcelona, Spain (I.S., R.E.)
[5] CIBER of Cardiovascular Diseases (CIBERCV), Barcelona, Spain (RE)

Table 1. Codes for identification of CHD incident events.

Condition	Primary Hospital Discharge Diagnosis ICD-9 Codes	CPT4 Codes	Primary Hospital Discharge Diagnosis Codes or Underlying Cause of Death ICD-10 Codes
Coronary Heart Disease			
Unstable and stable angina pectoris	411.1, 413.x		I20.x, I25.11x, I25.7x
Acute myocardial infarction	410.x		I21.x, I22.x
Coronary revascularization procedure	36.01, 36.02, 36.05, 36.06, 36.07, 36.09 36.10, 36.11, 36.12, 36.13, 36.14, 36.15, 36.16, 36.17, 36.19, 36.03	33510, 33511, 33512, 33513, 33514, 33515, 33516, 33517, 33518, 33519, 33521, 33522, 33523, 33530, 33533, 33534, 33535, 33536, 92980, 92981, 92982, 92984, 92995, 92996	
Coronary heart disease death			I11, I20-I22, I25

Table 2. Constituent SNPs for the Two Genetic Risk Scores.

**rsID**	**Gene loci**	**Chr**	**Risk/non-risk allele**	**RAF**	**p-HWE**	**GRS_12**	**GRS_51**	**Weights**
rs602633	SORT1	1	C/A	0.78	0.63		•	0.1133
rs17114036	PPAP2B	1	A/G	0.91	0.80		•	0.1044
rs4845625	IL6R	1	T/C	0.42	0.89		•	0.0488
rs11206510	PCSK9	1	T/C	0.82	0.85		•	0.0583
rs17464857	MIA3	1	T/G	0.85	1.00	•	•	0.0488
rs67258870*	WDR12	2	C/T	0.13	0.83	•	•	0.1133
rs515135*	APOB	2	G/A	0.81	1.00		•	0.077
rs2252641	ZEB2-ACO74093	2	G/A	0.45	0.89		•	0.0488
rs1561198	VAMP5-VAMP8- GGCX	2	A/G	0.46	1.00		•	0.0583
rs6544713*	ABCG5-ABCG8	2	T/C	0.32	0.88		•	0.0583
rs9818870*	MRAS	3	T/C	0.15	1.00	•	•	0.0677
rs7692387	GUCY1A3	4	G/A	0.81	1.00		•	0.0677
rs1878406	EDNRA	4	T/C	0.14	0.91		•	0.077
rs273909*	SLC22A4-SLC22A5	5	C/T	0.11	1.00		•	0.077
rs12190287*	TCF21	6	C/G	0.63	1.00		•	0.0677
rs10455872	SLC22A3-LPAL2-LPA	6	G/A	0.07	0.76	•	•	0.3000
rs2048327*	SLC22A3-LPAL2-LPA	6	G/A	0.36	1.00		•	0.0583
rs3798220	SLC22A3-LPAL2-LPA	6	C/T	0.02	0.61		•	0.2469
rs12526453	PHACTR1	6	C/G	0.66	0.77	•	•	0.0677
rs10947789	KCKN5	6	T/C	0.76	1.00		•	0.0677
rs4252120	PLG	6	T/C	0.71	0.70		•	0.0677
rs12205331	ANKS1A	6	C/T	0.80	1.00		•	0.0583
rs2023938	HDAC9	7	G/A	0.10	0.79		•	0.077
rs12539895	7q22	7	A/C	0.21	0.90		•	0.077
rs264	LPL	8	G/A	0.85	0.82		•	0.077
rs2954029	TRIB1	8	A/T	0.54	1.00		•	0.0488
rs1333049*	CDKN2B-AS1	9	C/G	0.49	1.00	•	•	0.207
rs3217992*	CDKN2B-AS1	9	A/G	0.38	0.78		•	0.1484
rs579459	ABO	9	C/T	0.21	0.62		•	0.0677
rs12413409	CYP17A1-CNNM2-NT5C2	10	G/A	0.91	1.00		•	0.0953
rs2505083	KIAA1462	10	C/T	0.42	0.86		•	0.0583
rs501120	CXCL12	10	A/G	0.86	0.81	•	•	0.0583
rs2047009	CXCL12	10	C/A	0.49	1.00		•	0.0488
rs2246833	LIPA	10	T/C	0.34	0.88		•	0.0583
rs974819	PDGFD	11	A/G	0.30	1.00		•	0.0677
rs9326246*	ZNF259-APOA5-APOA1	11	C/G	0.07	0.77		•	0.0862
rs3184504*	SH2B3	12	T/C	0.49	0.84		•	0.0677
rs9515203*	COL4A1-COL4A2	13	T/C	0.74	1.00		•	0.077
rs9319428	FLT1	13	A/G	0.30	1.00		•	0.0583
rs7173743	ADAMTS7	15	T/C	0.54	1.00		•	0.0677
rs12936587	RAI1-PEMT-RASD1	17	G/A	0.54	0.89		•	0.0583
rs2281727	SMG6	17	C/T	0.35	0.83		•	0.0488
rs15563	UBE2Z	17	C/T	0.53	1.00		•	0.0583
rs1122608	LDLR	19	G/T	0.76	0.83		•	0.0953
rs2075650*	APOE-APOC1	19	G/A	0.15	0.68		•	0.1044
rs445925	APOE-APOC1	19	C/T	0.89	0.31		•	0.1222
rs9982601*	KCNE2-SCL5A3	21	T/C	0.13	0.91	•	•	0.1222
rs10507391	ALOX5AP	13	A/T	0.33	1.00	•	•	0.131
rs17222842	ALOX5AP	13	G/A	0.92	0.91	•	•	
rs9315050	ALOX5AP	13	G/A	0.07	1.00	•	•	
rs17216473	ALOX5AP	13	G/A	0.13	0.91	•	•	
**Number of SNPs / Haplotypes**						**8 / 1**	**47 / 1**	

Chr: Chromosome; RAF: Risk allele frequency; p-HWE: p-value for the Hardy-Weinberg equilibrium test; Weigths: Beta values used to weight each variant within the GRS (values obtained from CardiogramPlusC4D consortium).
*SNPs directly genotyped.

Table 3. Rates and adjusted hazard ratios of incident CHD, GERA cohort, Minority Subjects (n=11,242).

Baseline Characteristics	Num. events (n=450)	Age-adjusted rate per 10,000 person years (SE)	Multivariable – adjusted hazard ratio (95% CI)†
Age, years			
30-54	11	7.1 (0.30)	1.00
55-64	159	50.4 (0.08)	3.90 (2.18 – 6.97)
65-79	211	94.6 (0.07)	5.48 (3.04 – 9.87)
Gender			
Male	212	57.4 (0.35)	1.74 (1.40 – 2.16)
Female	238	26.4 (0.35)	1.00
Race/Ethnicity			
African-American	95	36.4 (0.38)	1.37 (1.06 – 1.77)
Latino	316	34.8 (0.35)	1.12 (0.88 – 1.42)
Asian	39	37.2 (0.39)	1.00
Education level			
Less than college	120	47.7 (0.36)	0.94 (0.74 – 1.19)
College or higher	166	34.8 (0.36)	1.00
Missing	164	30.3 (0.35)	0.95 (0.68 – 1.33)
Smoking status			
Never	250	30.7 (0.35)	1.00
Former	164	42.3 (0.37)	1.08 (0.88 – 1.33)
Current	36	61.6 (0.39)	1.49 (1.04 – 2.14)
Alcohol consumption			
Abstinence	250	35.8 (0.35)	1.45 (1.04 – 2.02)
Light	94	34.8 (0.36)	1.24 (0.86 – 1.79)
Moderate	44	22.8 (0.38)	1.00
Heavy	14	67.2 (0.44)	2.69 (1.46 – 4.93)
Missing	48	54.5 (0.38)	1.95 (1.29 – 2.96)
Diabetes mellitus			
No	258	27.6 (0.35)	1.00
Yes	192	67.9 (0.37)	1.32 (1.06 – 1.64)
Body mass index, kg/m^2			
< 18	3	28.9 (0.67)	1.25 (0.39 – 3.94)
18 – 24.9	114	23.0 (0.36)	1.00
25 – 29.9	168	40.2 (0.36)	1.09 (0.84 – 1.40)
>=30	131	45.8 (0.36)	1.00 (0.75 – 1.33)
Missing	34	53.8 (0.39)	1.59 (1.07 – 2.37)
Anti-hypertensives, n (%)			
No	97	17.5 (0.35)	1.00
Yes	353	61.8 (0.38)	2.23 (1.73 – 2.88)
Total cholesterol, mg/dL			
< 200	297	40.8 (0.35)	1.00
200-239	114	29.0 (0.35)	1.14 (0.91 – 1.44)
≥ 240	39	25.8 (0.38)	1.00 (0.71 – 1.43)
HDL-C mg/dL			
< 35	38	73.4 (0.38)	1.51 (1.04 – 2.18)
35-45	132	55.6 (0.35)	1.22 (0.93 – 1.60)
45-55	135	37.4 (0.35)	1.17 (0.91 – 1.50)
≥ 55	145	23.1 (0.35)	1.00

Cholesterol lowering drugs, n (%)			
No	157	23.1 (0.35)	1.00
Yes	293	61.1 (0.38)	1.62 (1.29 – 2.03)
Estimated GFR, mL/min/1.73 m^2			
< 60	107	130.1 (0.10)	0.97 (0.75 – 1.25)
60-90	262	42.5 (0.06)	1.68 (1.23 – 2.28)
>90	80	31.7 (0.11)	1.00
Missing	1	3.3 (1.00)	0.31 (0.04 – 2.25)
Family History of Angina/heart attack			
No	303	31.1 (0.35)	1.00
Yes	132	45.5 (0.36)	1.41 (1.15 – 1.74)
Missing	15	86.3 (0.43)	1.88 (1.04 – 3.39)
Framingham Risk Score (%)*			
Low (< 10%)	208	27.7 (0.07)	1.00
Intermediate (10-20%)	186	100.0 (0.07)	3.62 (2.97 – 4.41)
High (< 20%)	56	129.2 (0.13)	4.59 (3.41 – 6.18)

*separate model with only intermediate and high Framingham risk score as independent variables.

Table 4a. Reclassification of Incident CHD risk after including GRS among African-Americans (n=2,089).

GRS_12

NRI table for expected events by Kaplan-Meier

Model without GRS12	Model with GRS12			
	Low <10%	Intermediate Low 10-15%	Intermediate High 15-20%	High >20%
Low <0.10%	28	6	0	0
Intermediate Low 10-15%	4	21	4	0
Intermediate High 15-20%	0	1	8	4
High >20%	0	0	0	11

NRI table for expected non-events by Kaplan-Meier

Model without GRS12	Model with GRS12			
	Low <10%	Intermediate Low 10-15%	Intermediate High 15-20%	High >20%
Low <0.10%	1393	69	1	0
Intermediate Low 10-15%	62	197	38	7
Intermediate High 15-20%	0	34	73	22
High >20%	0	1	22	86

GRS_51

NRI table for expected events by Kaplan-Meier

Model without GRS51	Model with GRS51			
	Low <10%	Intermediate Low 10-15%	Intermediate High 15-20%	High >20%
Low <0.10%	32	2	0	0
Intermediate Low 10-15%	3	25	1	0
Intermediate High 15-20%	0	1	9	3
High >20%	0	0	0	10

NRI table for expected non-events by Kaplan-Meier

Model without GRS51	Model with GRS51			
	Low <10%	Intermediate Low 10-15%	Intermediate High 15-20%	High >20%
Low <0.10%	1424	39	0	0
Intermediate Low 10-15%	27	253	23	0
Intermediate High 15-20%	0	17	103	9
High >20%	0	0	13	96

Table 4b. Reclassification of Incident CHD risk after including GRS among Latinos (n=4,349).

GRS_12

NRI table for expected events by Kaplan-Meier

Model without GRS12	Model with GRS12			
	Low <10%	Intermediate Low 10-15%	Intermediate High 15-20%	High >20%
Low <0.10%	52	5	0	0
Intermediate Low 10-15%	2	17	8	0
Intermediate High 15-20%	0	5	10	6
High >20%	0	0	3	13

NRI table for expected non-events by Kaplan-Meier

Model without GRS12	Model with GRS12			
	Low <10%	Intermediate Low 10-15%	Intermediate High 15-20%	High >20%
Low <0.10%	3094	110	1	0
Intermediate Low 10-15%	123	385	89	3
Intermediate High 15-20%	0	57	149	22
High >20%	0	0	35	160

GRS_51

NRI table for expected events by Kaplan-Meier

Model without GRS51	Model with GRS51			
	Low <10%	Intermediate Low 10-15%	Intermediate High 15-20%	High >20%
Low <0.10%	52	5	0	0
Intermediate Low 10-15%	3	20	4	0
Intermediate High 15-20%	0	3	10	9
High >20%	0	0	4	12

NRI table for expected non-events by Kaplan-Meier

Model without GRS51	Model with GRS51			
	Low <10%	Intermediate Low 10-15%	Intermediate High 15-20%	High >20%
Low <0.10%	3084	121	0	0
Intermediate Low 10-15%	123	391	86	0
Intermediate High 15-20%	0	54	144	30
High >20%	0	2	27	166

Table 4c. Reclassification of Incident CHD risk after including GRS among Asians (n=4,804).

GRS_12

NRI table for expected events by Kaplan-Meier

Model without GRS12	Model with GRS12			
	Low <10%	Intermediate Low 10-15%	Intermediate High 15-20%	High >20%
Low <0.10%	41	4	0	0
Intermediate Low 10-15%	1	26	3	0
Intermediate High 15-20%	0	0	14	2
High >20%	0	0	0	15

NRI table for expected non-events by Kaplan-Meier

Model without GRS12	Model with GRS12			
	Low <10%	Intermediate Low 10-15%	Intermediate High 15-20%	High >20%
Low <0.10%	3680	66	0	0
Intermediate Low 10-15%	57	479	31	0
Intermediate High 15-20%	0	43	157	17
High >20%	0	0	12	156

GRS_51

NRI table for expected events by Kaplan-Meier

Model without GRS51	Model with GRS51			
	Low <10%	Intermediate Low 10-15%	Intermediate High 15-20%	High >20%
Low <0.10%	39	6	0	0
Intermediate Low 10-15%	4	23	3	0
Intermediate High 15-20%	0	1	13	2
High >20%	0	0	0	15

NRI table for expected non-events by Kaplan-Meier

Model without GRS51	Model with GRS51			
	Low <10%	Intermediate Low 10-15%	Intermediate High 15-20%	High >20%
Low <0.10%	3665	81	0	0
Intermediate Low 10-15%	79	436	52	0
Intermediate High 15-20%	0	54	141	22
High >20%	0	0	20	148

Table 4d. Reclassification of Incident CHD risk after including GRS among All Minority Groups Combined (n=11,242).

GRS_12

NRI table for expected events by Kaplan-Meier

Model without GRS12	Model with GRS12			
	Low <10%	Intermediate Low 10-15%	Intermediate High 15-20%	High >20%
Low <0.10%	124	12	0	0
Intermediate Low 10-15%	8	68	9	0
Intermediate High 15-20%	0	3	36	11
High >20%	0	0	1	41

NRI table for expected non-events by Kaplan-Meier

Model without GRS12	Model with GRS12			
	Low <10%	Intermediate Low 10-15%	Intermediate High 15-20%	High >20%
Low <0.10%	8217	197	0	0
Intermediate Low 10-15%	194	1143	132	2
Intermediate High 15-20%	0	114	413	46
High >20%	0	0	53	418

GRS_51

NRI table for expected events by Kaplan-Meier

Model without GRS51	Model with GRS51			
	Low <10%	Intermediate Low 10-15%	Intermediate High 15-20%	High >20%
Low <0.10%	130	6	0	0
Intermediate Low 10-15%	5	76	5	0
Intermediate High 15-20%	0	3	41	7
High >20%	0	0	1	41

NRI table for expected non-events by Kaplan-Meier

Model without GRS51	Model with GRS51			
	Low <10%	Intermediate Low 10-15%	Intermediate High 15-20%	High >20%
Low <0.10%	8319	95	0	0
Intermediate Low 10-15%	106	1293	71	0
Intermediate High 15-20%	0	65	485	23
High >20%	0	0	33	438
